# Naturally evolved enhanced Cd tolerance of *Dianthus carthusianorum* L. is not related to accumulation of thiol peptides and organic acids

**DOI:** 10.1007/s11356-014-3963-8

**Published:** 2014-12-17

**Authors:** Małgorzata Wójcik, Sławomir Dresler, Andrzej Plak, Anna Tukiendorf

**Affiliations:** 1 Department of Plant Physiology, Faculty of Biology and Biotechnology, Maria Curie-Skłodowska University, Akademicka 19, 20-033 Lublin, Poland; 2 Department of Soil Science and Soil Protection, Faculty of Earth Sciences and Spatial Management, Maria Curie-Skłodowska University, Kraśnicka av 2 CD, 20-718 Lublin, Poland

**Keywords:** Cd tolerance, *Dianthus carthusianorum*, Glutathione, Organic acids, Phytochelatins, Proline

## Abstract

Two contrasting ecotypes of *Dianthus carthusianorum* L., metallicolous (M) and nonmetallicolous (NM), were cultivated in hydroponics at 0–50 μM Cd for 14 days to compare their Cd accumulation, sensitivity and tolerance mechanisms. While both ecotypes contained similar concentrations of Cd in the shoots and roots, the M ecotype was more Cd-tolerant (as measured by fresh weight production and root and leaf viability). Both ecotypes accumulated phytochelatins (PCs) in response to Cd with a higher amount thereof found in the NM ecotype. Concentrations of PCs remained unchanged with increasing Cd concentrations in the root tissues, but their content in the shoots increased. The addition of l-buthionine-sulfoximine (BSO) diminished glutathione (GSH) accumulation and arrested PC production, which increased the sensitivity to Cd of the NM, but not M ecotype. Organic acids (malate and citrate) as well as proline accumulation did not change significantly after Cd exposition and was at the same level in both ecotypes. The enhanced Cd tolerance of the M ecotype of *D. carthusianorum* cannot be explained in terms of restricted Cd uptake and differential production of PCs, organic acids or proline; some other mechanisms must be involved in its adaptation to the high Cd content in the environment.

**Electronic supplementary material:**

The online version of this article (doi:10.1007/s11356-014-3963-8) contains supplementary material, which is available to authorized users.

## Introduction

Environmental pollution with cadmium (Cd) poses a big threat due to the high mobility, persistence and toxicity of this element for all living organisms (Sanitá di Toppi and Gabbrielli 1999). Cadmium occurs naturally with Zn deposits; therefore, the Cd content in waste deposits left over mining and processing of Zn–Pb ores is particularly high. Such waste dumps represent an extremely harsh environment for establishment of vegetation; nevertheless, some plant species, belonging to the so-called ‘pseudometallophytes’, spontaneously colonize such areas. These plants usually represent common plant species but exhibit a greater ability to resist, tolerate or thrive in toxic metalliferous habitats compared with members of the same species from unpolluted soils (Whiting et al. 2004; Wójcik et al. 2014). This can be attributed to the development of a variety of mechanisms enabling them to avoid metal uptake or translocation within a plant as well as to detoxify the metals within their tissues. The latter mechanisms are mainly achieved by metal complexation with cellular ligands (glutathione (GSH), phytochelatins (PCs), organic acids and amino acids) and metal compartmentation at cellular and subcellular levels (Clemens 2001; Maestri et al. 2010).

Phytochelatins are small metal-binding peptides with the structure (γ-Glu-Cys)_*n*_-Gly in which *n* usually varies from 2 to 5, with maximum 11 (Pal and Rai 2010). They are synthesized from GSH (γ-Glu-Cys-Gly) by the enzyme γ-Glu-Cys dipeptidyl transpeptidase (PC synthase, EC 2.3.2.15), and the synthesis of both thiol compounds can be inhibited by l-buthionine-sulfoximine (BSO) which blocks γ-Glu-Cys formation. Apart from being the precursor of PC synthesis, GSH itself is also a potent chelator of Cd ions in the cytoplasm and a donor of these ions to PCs, although the existence of Cd-(GS)_2_ complexes in vivo has not been proven so far (Wójcik and Tukiendorf 2011 and ref. therein). On the other hand, the Cd-PC complexes have been found in many plant species, including, among others, *Silene vulgaris* (de Knecht et al. 1994), *Avena sativa* (Salt and Rauser 1995), *Brassica juncea* (Salt et al. 1995), *Thlaspi caerulescens* (Küpper et al. 2004), and *Arabidopsis thaliana* (Sadi et al. 2008). There is convincing evidence that PCs chelate Cd ions in the cytosol and transfer them into the vacuole, where more stable Cd-PC complexes are formed after incorporation of acid-labile sulphide (Sanità di Toppi and Gabbrielli 1999; Schat et al. 2002; Pal and Rai 2010, and ref. therein). Although there is no doubt about the role of PCs in Cd detoxification and compartmentalization, the contribution of PCs to Cd tolerance is still controversial. Some authors have suggested that increased Cd tolerance is associated with higher PC accumulation (Dresler et al. 2014). On the contrary, others have found no positive correlation between PC production and enhanced Cd tolerance (Schat et al. 2002; Wójcik et al. 2005; Wójcik et al. 2006; Zhang et al. 2010; Fernández et al. 2014b).

The role of organic acids and amino acids in plant Cd tolerance is even more questionable. Organic acids (malate and citrate) have been proved to contribute to the metal chelation for detoxification (mainly inside the vacuole) (Wang et al. 1991; Ueno et al. 2005) and root-to-shoot translocation (Wei et al. 2007). However, studies on the composition and pattern of accumulation of different organic acids in various plant species following Cd exposure have provided contrasting results (Boominathan and Doran 2003; Küpper et al. 2004; Ueno et al. 2005; Sun et al. 2006; Wójcik et al. 2006; Sun et al. 2011; Fernández et al. 2014a). Two amino acids, histidine and proline, have been found to form complexes with Cd in vivo (Schat et al. 1997; Sharma et al. 1998; Küpper et al. 2004). Nonetheless, no clear-cut evidence regarding their role in Cd detoxification and tolerance is available (Küpper et al. 2004; Ueno et al. 2005).

Plant species differ significantly in terms of metal accumulation and tolerance; substantial differences in these characteristics and underlying mechanisms have also been found between ecotypes/populations of the same species (Deng et al. 2007; Olko et al. 2008; Fernández et al. 2013; Dresler et al. 2014; Wójcik and Tukiendorf 2014). Therefore, the investigations at the species level provide an excellent opportunity to unravel the mechanisms behind plant adaptation to adverse mineral conditions, also in terms of choosing the best plant species/ecotypes for revegetation or remediation of contaminated sites.

*Dianthus carthusianorum* L. belongs to species that can grow well in both uncontaminated and metal-contaminated areas (Wójcik et al. 2013). It is also one of the dominant species on the over 100-year-old waste deposit formed of by-products of Zn–Pb ore mining and smelting in Bolesław, southern Poland. Our previous studies revealed that a metallicolous ecotype of *D. carthusianorum* demonstrated different morphological features and molecular marker signature as well as higher Pb tolerance in comparison with a nonmetallicolous ecotype (Wójcik et al. 2013; Wójcik and Tukiendorf 2014). However, to the best of our knowledge, Cd tolerance and the mechanisms of Cd detoxification have scarcely been studied in this species so far (Załęcka and Wierzbicka 2002). The objective of the present study was to examine whether the metallicolous ecotype of *D. carthusianorum* exhibits higher tolerance to Cd as well and if the tolerance is related to Cd accumulation and enhanced production of PCs, organic acids and proline. Moreover, in order to assess the role of PCs in Cd tolerance, we tested how BSO-induced diminishing of GSH and PC synthesis affect the plant response to Cd.

## Material and methods

### Plant material and growth conditions

Two ecotypes of *D. carthusianorum* L. (Caryophyllaceae) (Carthusian pink) were analyzed in this study. The metallicolous (M) ecotype originated from a Zn–Pb waste deposit in Bolesław, near Olkusz, southern Poland, and the nonmetallicolous (NM) ecotype originated from an uncontaminated soil in Pliszczyn, near Lublin, south-eastern Poland, as described previously (Wójcik et al. 2013). The seeds collected in bulk from the plants growing in their natural habitats germinated for a week in a commercial garden soil (doubly autoclaved), and the seedlings were transplanted on the same soil for further 6-week growth. Uniformly sized plants were then transferred by one into pots filled with 0.5 L of full strength Hoagland’s nutrient solution (pH app. 5.6). Soil and hydroponic cultures were conducted in a growth room at 24/18 °C (day/night) with a 16-h photoperiod (150 μmol m^−2^ s^−1^) and humidity of 60 %.

After a 2-day acclimation to the hydroponic culture, the plants were treated with 0, 5, 15 or 50 μM Cd (in the form of Cd(NO_3_)_2_ × 4 H_2_O) added to the fresh nutrient solution. In order to determine the effect of BSO on GSH/PC synthesis and plant Cd tolerance, additional treatments with 0, 50 or 250 μM BSO added to 0 or 50 μM Cd-supplemented nutrient solutions were applied. Solutions were continuously aerated and replaced weekly. The plants were analyzed after 14 days of Cd and/or BSO treatment.

### Cadmium and mineral nutrient concentrations in plants

The roots were washed in an ice-cold 10 mM CaCl_2_ solution for 30 min to enable cation exchange between Ca^2+^ and Cd^2+^ adsorbed to the root surface. Root and shoot samples were subsequently washed in distilled water, and subsamples of 0.2–0.4 g were dried at 105 °C to a constant weight and wet digested in HNO_3_/HClO_4_ (4:1, *v*/*v*) using a Velp Scientica digester, DK20 (Milan, Italy). The concentrations of Cd as well as Zn, Mg, Fe, Mn and Cu were determined by atomic absorption spectrophotometry (AAS, Perkin–Elmer, Model 3300, Waltham, Massachusetts, USA) using Fluka standard solutions. The analyses were performed in 3–4 replications (from 3–4 independent time series per treatment in each ecotype, a composite sample of roots or shoots of five plants from each time series was used as a replication).

### Plant growth and the viability of roots and shoots

The fresh (FW) and dry (DW) weight of plant roots and shoots was determined. The water content in plant tissues was calculated using the formula (FW − DW)/FW and expressed as the percentage of the FW. Data for fresh weight assays were obtained from 30–70 individual plants per treatment in each ecotype from 6–10 independent time series in total. Dry weight and water content were analyzed in four composite samples of roots and shoots (a mixture of five plants in each) per treatment in each ecotype.

Root viability was analyzed by staining root tips with a fluorescein diacetate-propidium iodide (FDA-PI) mixture as described by Ishikawa and Wagatsuma (1998). The images were observed under a light microscope equipped with epi-fluorescence mode (Olympus BX41, Hamburg, Germany) (excitation at 460–495 nm, emission at 510 nm) and recorded using an Olympus-Camedia C-5060 digital photo camera and analySIS software (Soft Imagin System GmbH, Münster, Germany).

Leaf viability was detected histochemically by trypan blue staining according to the method described by Joo et al. (2005) with slight modifications. The images were recorded under a light microscope as described above. The images of root and leaf viability were collected from at least ten roots or leaves per treatment in each ecotype using five different plants.

### Content of photosynthetic pigments and anthocyanins

For determination of photosynthetic pigments and anthocyanins, shoot samples frozen in liquid nitrogen and stored at −80 °C prior to analysis were used. Chlorophyll and carotenoids were extracted using 80 % acetone and determined according to the method described by Wellburn (1994). The total anthocyanin content expressed as absorbance units corrected by chlorophyll contribution (A530-0.25*A657) was determined using the protocol described by Hawrylak-Nowak (2008). Five shoot samples (composed of a mixture of leaves of five different plants per sample) per treatment in each ecotype were analyzed.

### Thiol peptides, organic acids and proline

Fresh root and shoot samples were used for assays of thiol peptides, organic acids and free proline. Three to four or five composite samples (a mixture of roots or leaves of five plants in each) from 3–5 independent time series per treatment in each ecotype were used for analyses of thiol peptides and organic acids or proline, respectively.

Thiol peptides were determined by HPLC with post-column reaction with 5,5′-dithiobis-2-nitrobenzoic acid (DTNB), as described previously (Wójcik et al. 2005). The chromatograms were recorded and analyzed using 32 Karat 7.0 software (Beckman). Standards of GSH (Sigma-Aldrich, St. Louis, MO, USA) and PCs (*n* = 2–4, Anaspec Inc., San Jose, CA, USA) were used to identify peaks on the chromatograms obtained.

Organic acids were analyzed spectrophotometrically using enzymatic test kits (Boehringer, Mannheim, Germany) as described by Olko et al. (2008). The free proline content was determined using a colorimetric method as described previously (Dresler et al. 2014).

### Statistical analysis

Non-parametric Kruskal-Wallis and Mann-Whitney *U* tests were used to determine statistical significance of the differences between the Cd treatments within the ecotypes as well as between the ecotypes at the same Cd level. All analyses were performed using Statistica 8.0 software (StatSoft, Inc.), and statistical significance of the analyses was defined at *p* < 0.05.

## Results

### Cd accumulation and mineral composition

**Fig. 1:**
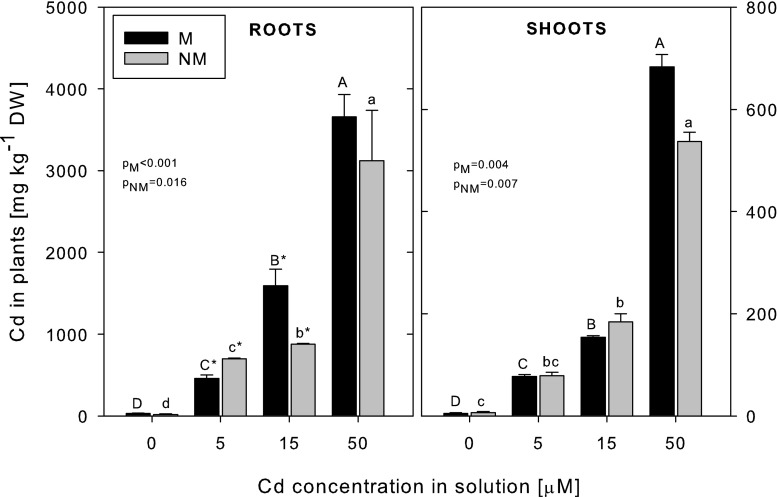
The concentrations of Cd in metallicolous (*M*) and nonmetallicolous (*NM*) plants of *D. carthusianorum* exposed to varying Cd concentrations for 14 days. Means ± SE. *p* values of Kruskal-Wallis analyses are given. The values followed by the *same letters* (*upper case letters* for M ecotype and *lower case letters* for NM ecotype) are not significantly different at *p* < 0.05. *Asterisks* indicate significant differences between the ecotypes at the same Cd treatments (*p* < 0.05)

The Cd concentration increased in the roots and shoots of both ecotypes with the increasing Cd concentration in the nutrient medium (Fig. 1). The metal concentrations in the roots were always higher than in the shoots (by 5–10 times), and at the highest Cd treatment, the shoot/root translocation factor was similar in both ecotypes (0.19 and 0.17 for the M and NM ecotypes, respectively). At 50 μM Cd, the difference in Cd accumulation in the roots between the ecotypes was not statistically significant; however, at 5 μM Cd, the NM plants accumulated more Cd, but in contrast, at 15 μM Cd, the M plants accumulated more Cd in their roots than the NM plants. The Cd concentrations in the shoots were similar in the plants of both ecotypes at each Cd exposure level (*p* > 0.05).

The concentrations of Zn, Mg, Fe and Cu in the roots and shoots were not influenced by any Cd treatment and were similar in both ecotypes (data not shown). Only the Mn concentration decreased significantly (by 56–66 %) in the roots and, to a lesser extent, in the shoots (by 39–44 %) of Cd-treated plants, and it was higher in the shoots of the NM ecotype (data not shown).

### Cd toxicity on plant growth and viability

**Fig. 2:**
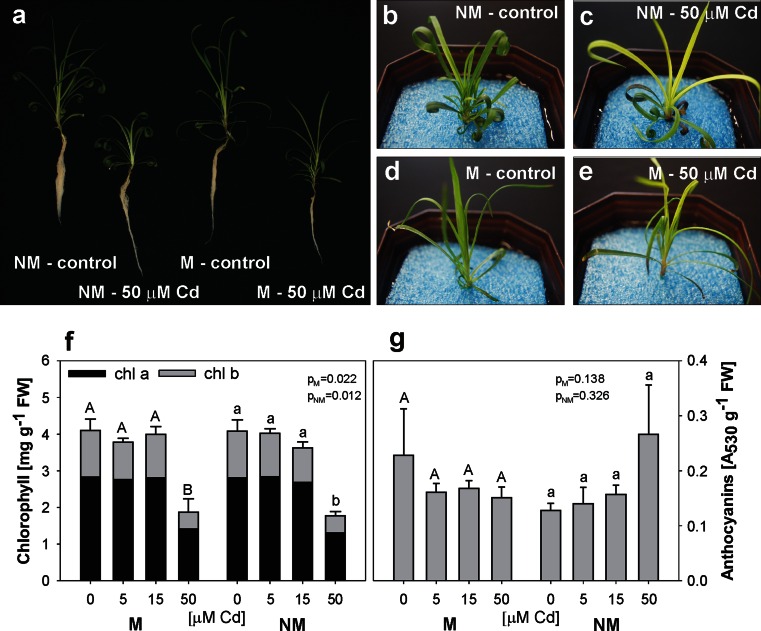
General appearance (**a**–**e**) and concentration of chlorophylls (**f**) and anthocyanins (**g**) in metallicolous (*M*) and nonmetallicolous (*NM*) plants of *D. carthusianorum* cultivated for 14 days in the control conditions or in the presence of Cd. *p* values of Kruskal-Wallis analyses are given in **f**–**g**. The values followed by the *same letters* (*upper case letters* for M ecotype and *lower case letters* for NM ecotype) are not significantly different at *p* < 0.05. There were no significant differences between the ecotypes

Cadmium induced visible symptoms of phytotoxicity, such as reduced growth, chlorosis of younger leaves and purple colour of the oldest leaves (Fig. 2a–e). The visible leaf discolorations (Fig. 2c, e) were confirmed by quantitative analysis of chlorophyll but not anthocyanin concentrations (Fig. 2f–g). The reduction of the content of chlorophylls was significant only at the highest Cd exposure (by app. 50 and 64 % for chl *a* and chl *b*, respectively), and it was similar in both ecotypes (no significant differences between the ecotypes) (Fig. 2f). However, the anthocyanin concentration did not change significantly at any Cd treatment (Fig. 2g).

**Fig. 3:**
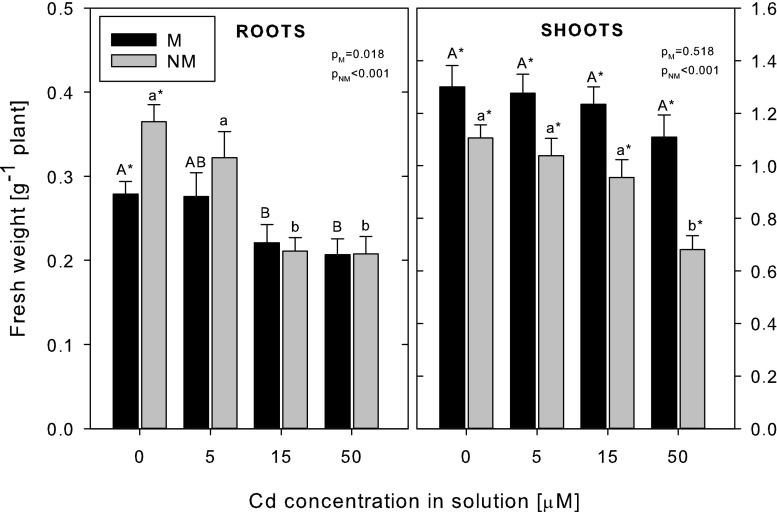
Fresh weight of metallicolous (*M*) and nonmetallicolous (*NM*) plants of *D. carthusianorum* exposed to varying Cd concentrations for 14 days. Means ± SE (*n* = 30–90). The values followed by the *same letters* (*upper case letters* for M ecotype and *lower case letters* for NM ecotype) are not significantly different at *p* < 0.05. *Asterisks* indicate significant differences between the ecotypes at the same Cd treatments (*p* < 0.05). *p* values of Kruskal-Wallis analyses are given

The control (non-Cd-treated) plants of both ecotypes differed in terms of their fresh weight, with their root biomass being higher in the NM, but shoot biomass being higher in the M plants (Fig. 3). The Cd treatment significantly reduced the fresh weight of roots and shoots of the NM ecotype. The reduction of root fresh weight was observed at 15 and 50 μM Cd (by 42–43 %) and that of shoots only at 50 μM Cd (by 38 %) in comparison with the control plants. The fresh biomass of the roots of the M plants decreased by 21–26 % at 15–50 μM Cd, but in the shoots, it remained unchanged even at the highest Cd treatment. There were no significant differences in the dry weight and water content of the control and Cd-treated plants of both ecotypes (data not shown). The EC100 parameter was found to be 800 and 450 μM Cd in the M and NM plants, respectively.

Metal toxicity also affected the plasma membrane permeability, as shown by root and shoot viability assays (Supplementary Fig. 1 and Supplementary Fig. 2). Increasing Cd concentrations induced loss of root cell membrane stability in both ecotypes (visible as increasing contribution of red fluorescence over green fluorescence); however, it was much more pronounced in the NM ecotype (Supplementary Fig. 1), testifying to its higher sensitivity to Cd. Similarly, enhanced accumulation of trypan blue in the leaf cells, demonstrating increased cell membrane permeability, was observed with increased Cd concentrations in both ecotypes, however with considerably higher intensity in the NM ecotype (Supplementary Fig. 2).

### Accumulation of potential Cd-chelating ligands

**Fig. 4:**
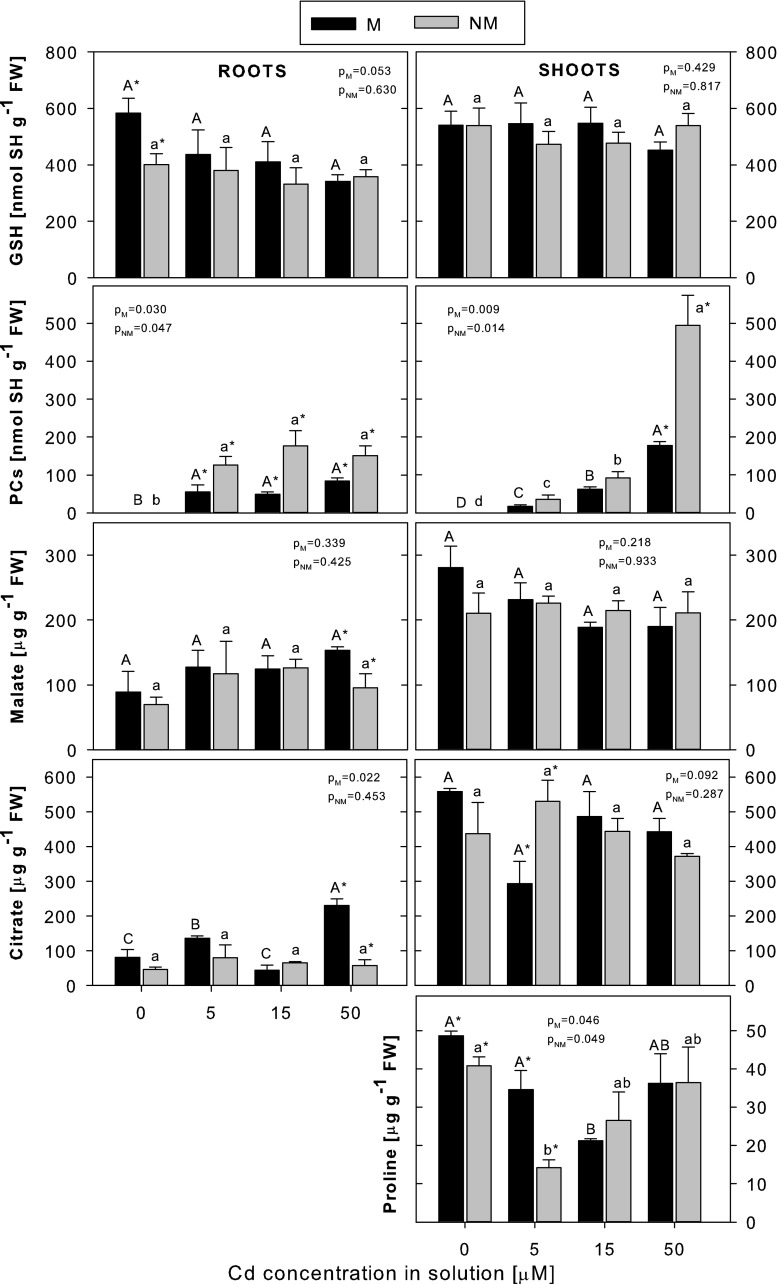
The concentrations of GSH, PCs, malate, citrate and proline in metallicolous (*M*) and nonmetallicolous (*NM*) plants of *D. carthusianorum* exposed to varying Cd concentrations for 14 days. Means ± SE. *p* values of Kruskal-Wallis analyses are given. The values followed by the *same letters* (*upper case letters* for M ecotype and *lower case letters* for NM ecotype) are not significantly different at *p* < 0.05. *Asterisks* indicate significant differences between the ecotypes at the same Cd treatments (*p* < 0.05)

Different kinds of potential Cd-chelating ligands: thiol peptides (GSH and PC), organic acids (malate and citrate) and the amino acid proline have been investigated in this study in relation to *D. carthusianorum* Cd tolerance (Fig. 4).

The GSH level was higher in the non-Cd-treated roots of the M ecotype than in the NM one, but in both ecotypes, the GSH concentrations remained unchanged and were similar to each other following the Cd treatment. There were no significant differences between the two ecotypes in the concentration of GSH in the shoots either, both in the control and Cd-treated plants; the amount of GSH also remained stable regardless of the Cd exposure level.

Cadmium induced PC accumulation in the roots and shoots of both ecotypes. While the PC concentrations in the roots did not change within the whole range of Cd exposure, in the shoots, a progressive increase in PC production with the increasing Cd exposure level was noted in both ecotypes. Interestingly, higher PC accumulation was found in both the roots (at 5–50 μM Cd) and shoots (at 50 μM Cd) of the NM ecotype.

The accumulation of both organic acids studied, i.e. malate and citrate, in general, did not vary with concentrations of Cd in either roots or shoots and did not differ between the ecotypes, with the exception of malate and citrate concentrations in the roots at 50 μM Cd, being higher in the M plants. The citrate content in the roots of the M plants was then also threefold higher than in the control plants.

The free proline content was determined only in plant shoots. For reasons that are unclear, it decreased at the lower Cd concentrations (at 5 or 15 μM Cd in the NM or M plants, respectively), but at the higher Cd concentrations, it remained at the control level. At the same time, in the control (0 μM Cd) or 5 μM Cd-treated plants, proline accumulation was significantly higher in the M ecotype; however, at higher Cd concentrations, there were no differences between the two ecotypes.

### Effect of BSO on production of thiol compounds and plant response to Cd

**Fig. 5:**
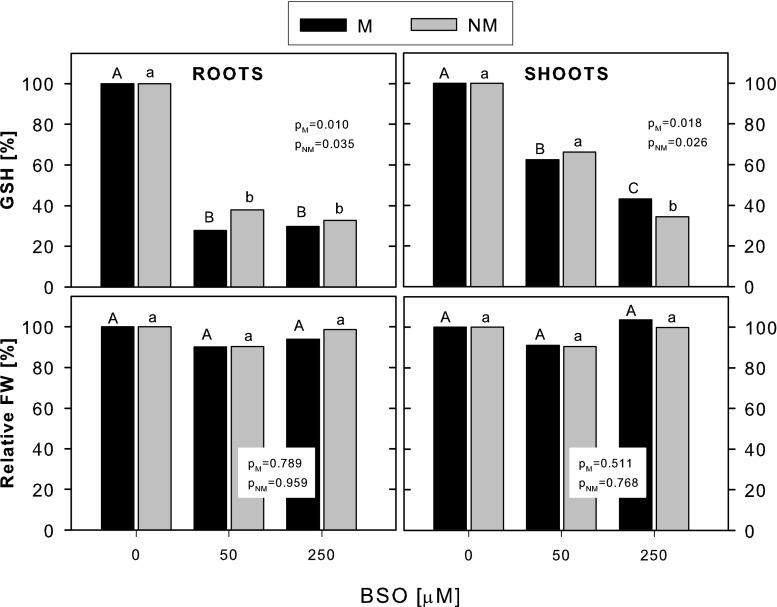
The effect of BSO on the accumulation of GSH and fresh weight of metallicolous (*M*) and nonmetallicolous (*NM*) plants of *D. carthusianorum* cultivated for 14 days in the control conditions (0 μM Cd). Data represent relative values calculated from the means of absolute values. The absolute values for GSH accumulation and for FW were analyzed statistically, and *p* values of Kruskal-Wallis analyses are given. The values followed by the *same letters* (*upper case letters* for M ecotype and *lower case letters* for NM ecotype) are not significantly different at *p* < 0.05

The addition of BSO strongly inhibited the synthesis of GSH in the non-Cd-treated plants, and the extent of this inhibition was similar in both ecotypes (Fig. 5). In the roots, the GSH concentration was reduced by ca. 62–70 % at 50 and 250 μM BSO exposure, and in the shoots, the reduction reached ca. 37 and 60 % at 50 and 250 μM BSO, respectively, compared with the respective control plants. Simultaneously, BSO did not influence the fresh weight of the roots and shoots of the non-Cd-treated plants of both ecotypes (Fig. 5), although a slight toxic effect of 250 μM BSO on root apex viability could be noticed (data not shown).

**Fig. 6:**
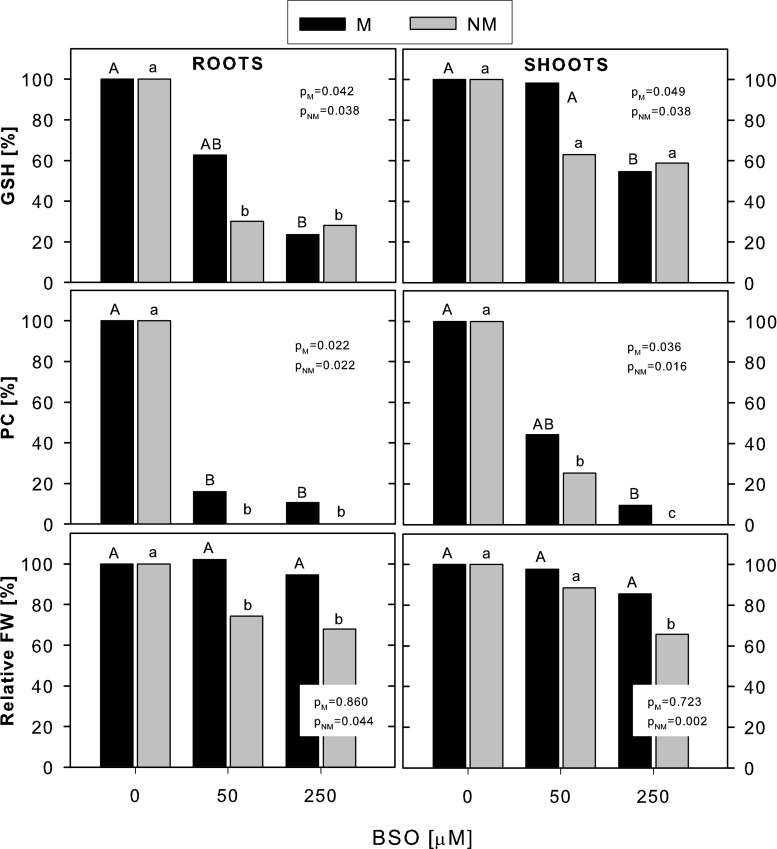
The effect of BSO on the accumulation of GSH, PC and fresh weight of metallicolous (*M*) and nonmetallicolous (*NM*) plants of *D. carthusianorum* exposed to 50 μM Cd for 14 days. Data represent relative values calculated from the means of absolute values. The absolute values for GSH and PC accumulation and for FW were analyzed statistically, and *p* values of Kruskal-Wallis analyses are given. The values followed by the *same letters* (*upper case letters* for M ecotype and *lower case letters* for NM ecotype) are not significantly different at *p* < 0.05

As seen in the non-Cd-treated plants, the addition of BSO to the Cd-treated plants decreased the GSH concentration in the roots of both ecotypes (by app. 75 % at 250 μM BSO); however, a significant decrease in shoot GSH was only found in the M plants (by app. 40 % at 250 μM BSO exposure) (Fig. 6). At the same time, PC production was completely arrested in the roots and shoots of the NM ecotype and severely reduced in the roots and shoots of the M ecotype (by 90 % at 250 μM BSO exposure). Comparison of GSH and PC concentrations in the NM and M plants revealed no significant differences at any Cd + BSO treatment. BSO-imposed inhibition of GSH and PC synthesis was accompanied by a significantly enhanced reduction of fresh weight of the Cd-stressed plants only in the NM ecotype (by 35 % in both roots and shoots at 250 μM BSO compared with the non-BSO-exposed plants), whereas in the M ecotype, the fresh weight was not affected by any BSO dose.

## Discussion

### Cd tolerance versus Cd accumulation

Plants adapted to growth in metalliferous habitats usually exhibit higher metal tolerance than plants (even populations/ecotypes of the same species) from unpolluted areas when exposed to the same level of metal stress. This phenomenon has been well documented for many plant species, including, among others, *S. vulgaris* (de Knecht et al. 1994), *Armeria maritima* (Olko et al. 2008), *Sedum alfredii* (Sun et al. 2007), *Dittrichia viscosa* (Fernández et al. 2013), *Echium vulgare* (Dresler et al. 2014), and also *D. carthusianorum* (Załęcka and Wierzbicka 2002; Wójcik and Tukiendorf 2014). Our present results are consistent with the previous reports. Based on the EC100 parameter and fresh biomass production, the NM ecotype appeared to be more Cd-sensitive than the M ecotype. At similar Cd concentrations in the root and shoot tissues at 50 μM Cd treatment, the fresh weight of both organs was significantly reduced in the NM ecotype, as compared with the control plants, but remained unaffected in the shoots of the M ecotype. Similarly, at the highest Cd treatment, root and leaf viability was much more affected in the NM than in the M plants. However, accumulation of anthocyanins, often considered as a marker of metal-induced stress (Krupa et al. 1996), did not change following any Cd treatment and was similar in both ecotypes. Likewise, while considering the chlorophyll content, the NM ecotype did not appear to be more Cd-sensitive than the M one. Cadmium-imposed loss of chlorophyll, a typical symptom of Cd toxicity, was equal in both ecotypes, reflecting a similar Cd accumulation pattern in the leaves. Therefore, these results confirm our previous finding that anthocyanins or chlorophyll content is not a parameter distinguishing between sensitive and tolerant ecotypes (Wójcik et al. 2013), although this is not in agreement with the study of Fernández et al. (2013).

The pattern of the metal accumulation is species/ecotype specific and is additionally modified by exposure concentration and time. However, it is evident in a majority of reports that enhanced metal tolerance is not related to restricted metal uptake by metallicolous plants. On the contrary, in *E. vulgare* plants, higher Cd concentrations were accumulated in both the roots and shoots of the metallicolous populations than in the nonmetallicolous one (Dresler et al. 2014). On the other hand, in some plant species, higher Cd concentrations were found in the shoots of metallicolous ecotypes and in the roots of nonmetallicolous ones (Gadapati and Macfie 2006; Sun et al. 2006; Sun et al. 2007; Fernández et al. 2014a, b). In other species, no significant differences in Cd accumulation between metallicolous and nonmetallicolous populations under the same Cd treatments were found (Hernández-Allica et al. 2006; Fernández et al. 2013). This was also the case in the present study, where both ecotypes accumulated similar amounts of Cd in their shoots (over the whole range of Cd concentrations used) and in the roots (at 50 μM Cd, the concentration differentiating the sensitivity of M and NM plants to Cd).

Since the difference in Cd tolerance between the ecotypes cannot be explained by different Cd accumulation, it must be achieved through internal detoxification systems.

### Cd tolerance versus PC accumulation

Of all potential Cd-chelating ligands, PC synthesis and accumulation have probably been investigated most intensively in terms of Cd detoxification and tolerance. Many studies, including these on mutants, transgenic plants or other plants with altered PC synthesis, have confirmed that these peptides are the main factor for basal Cd tolerance (Verbruggen et al. 2009; Pal and Rai 2010; Wójcik and Tukiendorf 2011), but their role in enhanced Cd tolerance or hypertolerance is not evident (Wójcik et al. 2006; Ernst et al. 2008). Although in some Cd-tolerant (metallicolous) ecotypes/populations PC accumulation was found to be higher than in Cd-sensitive (nonmetallicolous) ones, e.g. in *E. vulgare* (Dresler et al. 2014) or in *D. viscosa* (Fernández et al. 2014a, b), most reports, including the present study, have shown the opposite tendency. At 50 μM Cd in the solution, the NM plants of *D. carthusianorum* had a higher concentration of PCs in both the roots and shoots; nevertheless, they experienced more Cd-induced stress than the M plants did. A similar phenomenon was observed in NM and M ecotypes of *S. alfredii* (Sun et al. 2007), *S. vulgaris* (de Knecht et al. 1994), in non-Cd-accumulating and accumulating *T. caerulescens* (Schat et al. 2002), and in Cd-sensitive *Thlaspi arvense* compared with a Cd hyperaccumulator *T. caerulescens* (Ebbs et al. 2002). However, in some species, PC accumulation was higher in the roots of the Cd-tolerant than Cd-sensitive plants in spite of similar Cd concentrations, suggesting that the elevated root PC content could contribute to the higher Cd tolerance (Gadapati and Macfie 2006; Fernández et al. 2014b). Rapid Cd chelation by PC at the root, i.e. the metal entry level, might diminish its toxicity to plants.

To reveal the role of PC in Cd tolerance further, BSO was added to the growth medium to suppress GSH and, consequently, PCs synthesis. As expected, both GSH and especially PC accumulation was strongly reduced, as previously observed in many other studies (Schat et al. 2002; Hernández-Allica et al. 2006; Wójcik and Tukiendorf 2011; Fernández et al. 2014b). As a result, an increase in Cd sensitivity of the NM plants, but not of the M plants of *D. carthusianorum*, was found, a pattern observed earlier in Cd-sensitive and tolerant ecotypes of *S. vulgaris*, *T. caerulescens* and *D. viscosa* (Schat et al. 2002; Hernández-Allica et al. 2006; Fernández et al. 2014b). It was not examined in this study whether this could result from different Cd accumulation in the BSO-treated NM and M plants; however, it was confirmed previously that BSO did not influence significantly Cd accumulation (Wójcik and Tukiendorf 2011) and definitely did not differentiate the Cd uptake pattern between plant ecotypes (Hernández-Allica et al. 2006). Overall, these results unquestionably indicate that PC may play a role in basic Cd tolerance of nonmetallicolous plants but are not responsible for enhanced Cd tolerance of populations from metalliferous sites.

Another argument against the role of PCs in elevated metal tolerance of metallophytes is the fact that hardly ever is PC accumulation found in plants growing in metal-enriched soils (Wójcik et al. 2005; Ernst et al. 2008 and ref. therein). Neither has it been found in plants of *D. carthusianorum* inhabiting Zn–Pb waste deposits (Wójcik, unpublished data). This has previously been discussed by Sanitá di Toppi and Gabbrielli (1999) and Maestri et al. (2010), who suggested that in plants chronically exposed to heavy metals, the adaptation mechanism based on PC production is unlikely to be evolved due to very high demand for energy required for sulphate reduction.

Sun et al. (2007) proposed that instead of PCs, GSH might play a role in Cd tolerance of mine population of *S. alfredii*. Accordingly, others have also reported an increase in the GSH concentration following Cd exposure (Olko et al. 2008); however, in the present study and in the studies of Dresler et al. (2014), Fernández et al. (2014), or Gadapati and Macfie (2006), this was not the case. The differences in GSH accumulation between metallicolous and nonmetallicolous ecotypes/populations were not evident or uniform as well. In the present study and investigations of *E. vulgare* (Dresler et al. 2014), there was no difference between the M and NM ecotypes. In other reports, at the same Cd exposure levels, roots of metallicolous populations exhibited a higher GSH content than those of nonmetallicolous ones, but in the shoots, the proportion was opposite (Sun et al. 2007; Olko et al. 2008; Fernández et al. 2014b). Nevertheless, the high GSH content seems to not contribute to protection against Cd toxicity (Ernst et al. 2008; Wójcik and Tukiendorf 2011).

From these studies, it clearly appears that the presence of thiol peptides (GSH and PCs) alone is not enough to confer the Cd tolerance of metallicolous plants.

### Cd tolerance versus accumulation of organic acids and proline

Organic acids, especially malate and citrate, were studied in terms of metal tolerance, vacuolar sequestration and long distance transport mainly in hyperaccumulators, where constitutively high concentrations thereof are usually found (Boominatan and Doran 2003; Ueno et al. 2005; Callahan et al. 2006; Verbruggen et al. 2009). Nonetheless, it has been argued that organic acids are not responsible for the extraordinary abilities of hyperaccumulators to accumulate and tolerate extremely high amounts of metals (Callahan et al. 2006; Verbruggen et al. 2009). Comparative studies of accumulation of organic acids have also been conducted in Cd-stressed species/populations originating from metalliferous and uncontaminated sites providing, however, contrasting results (Sun et al. 2006; Olko et al. 2008; Dresler et al. 2014; Fernández et al. 2014a). In agreement with our study, no significant differences in the malate and citrate concentrations were found in the metallicolous and nonmetallicolous populations of *A. maritima* (Olko et al. 2008) and *E. vulgare* (Dresler et al. 2014); however, malate strongly dominated in Cd-tolerant *Solanum nigrum* (Sun et al. 2006). In some Cd-treated plant species, an increase in malate was found, but in others, a rise in the citrate concentration was reported instead, suggesting or excluding their role in Cd tolerance (Sun et al. 2006; Olko et al. 2008). Our results do not support the importance of malate in *D. carthusianorum* response to Cd. The fact that the citrate concentration in the roots of the 50-μM Cd-treated plants increased and was higher in the M than NM ecotype may suggest its role in Cd detoxification, as was also suggested in the studies of Sun et al. (2006) and Fernández et al. (2014a); nonetheless, this does not strongly contradict the general conclusion against the role of organic acids in enhanced Cd tolerance.

Several reports have shown an increase in proline accumulation in Cd-stressed plants (Schat et al. 1997; Fernández et al. 2013), and some suggested a role of proline in cellular Cd detoxification (Sharma et al. 1998). Moreover, the constitutive proline concentration in leaves was proved to be higher in Cd-tolerant metallicolous ecotype of *S. vulgaris* than in the Cd-sensitive one, and this phenomenon was not related to differential Cd accumulation (Schat et al. 1997). Consistent with this report, the M plants of *D. carthusianorum* grown at 0–5 μM Cd had higher proline content in the shoots than the NM plants; however, this was not the case at the higher Cd concentrations. Similarly, no differences in proline accumulation between metallicolous and nonmetallicolous ecotypes of *D. viscosa* and *E. vulgare* were found regardless of the Cd level (Fernández et al. 2013; Dresler et al. 2014). In addition, our previous study did not show any differences in the proline content between the ecotypes of *D. carthusianorum* grown in their natural (metalliferous or unpolluted) habitats and on garden soil (Wójcik et al. 2013). Furthermore, in contrast to what was reported by Schat et al. (1997) and Fernández et al. (2013), in the present study, no significant change in proline accumulation was found following the Cd treatment in any ecotype, despite the increasing Cd content in the tissues. However, contrary to the two latter studies, we did not observe any disturbances in water balance induced by Cd, which is believed to be the main cause of a proline accumulation increase. In summary, our results do not support the role of proline in Cd detoxification and tolerance.

The mechanisms behind enhanced Cd tolerance of the M ecotype of *D. carthusianorum* should still be investigated and elucidated. Probably, the diverse Cd sensitivity of the M and NM ecotypes might be explained by a different Cd subcellular distribution pattern, as was found in the case of Pb toxicity in *D. carthusianorum* (Baranowska-Morek and Wierzbicka 2004). Increased capacity of the cell wall to bind substantial amounts of Cd or effective vacuolar sequestration would prevent the metal toxicity in the cytoplasm. The former might be achieved, e.g. by thickening of the cell wall due to increased amount of polysaccharides (Fernández et al. 2014a), and the latter by more efficient Cd chelation in the cytosol followed by fast translocation of the Cd-ligand (GSH or PC?) complexes into the vacuole and their stabilization by incorporation of labile sulphide at the same or even lower content of ligands in comparison with the NM ecotype (reviewed by Sanitá di Toppi and Gabbrielli 1999). Therefore, more advanced studies enabling recognition of Cd localization and speciation at tissue, cellular and subcellular levels should be continued.

## Conclusions

The present paper provides strong evidence that neither restricted Cd uptake and translocation nor elevated production of PC, organic acids and proline are involved in Cd tolerance of the metallicolous ecotype of *D. carthusianorum*. The following results support the above statement: (i) Cd accumulation and root-to-shoot translocation were similar in both ecotypes; nevertheless, the NM ecotype exhibited more pronounced Cd toxicity symptoms. (ii) Despite lower PC accumulation, the M ecotype showed higher tolerance to Cd; furthermore, it was unaffected by the BSO treatment, implying that PC synthesis is not a key determinant of enhanced Cd tolerance observed in this ecotype. (iii) In contrast, BSO addition increased Cd sensitivity of the NM ecotype, suggesting that PC-mediated Cd detoxification may play a certain role in this ecotype. (iv) The accumulation of organic acids and proline was in general unchanged by the Cd treatment and remained at a similar level in both ecotypes, testifying against a role of these compounds in both alleviation of Cd-induced stress or elevated Cd tolerance of the M ecotype. Therefore, further examinations are needed to understand fully the mechanisms underlying the enhanced Cd tolerance of the metallicolous plants/ecotypes, including *D. carthusianorum*.

## Electronic supplementary material

Below is the link to the electronic supplementary material.

Fig 1 (TIFF 17013 kb) Root viability of metallicolous (M) and nonmetallicolous (NM) plants of *D. carthusianorum* exposed to varying Cd concentrations for 14 days. Images of roots incubated in the mixture of FDA-PI were taken under fluorescent microscope at lens magnification 4x. Green fluorescence (metabolized FDA) and red fluorescence (PI) indicates viable and dead cells, respectively. Scale bars - 200 μm

Fig 2 (TIFF 16961 kb) Leaf viability of metallicolous (M) and nonmetallicolous (NM) plants of *D. carthusianorum* exposed to varying Cd concentrations for 14 days. Images of leaves stained in trypan blue were taken under light microscope at lens magnification 4×. Blue colour indicates cells with damaged plasma membranes. *Scale bars* - 200 μm
