# Supplementary figures and images for: Naturally evolved enhanced Cd tolerance of Dianthus carthusianorum L. is not related to accumulation of thiol peptides and organic acids
Source: Environ Sci Pollut Res Int. 2014 Dec 17;22(10):7906–17. doi: 10.1007/s11356-014-3963-8 (PMC4432087; doi:10.1007/s11356-014-3963-8)

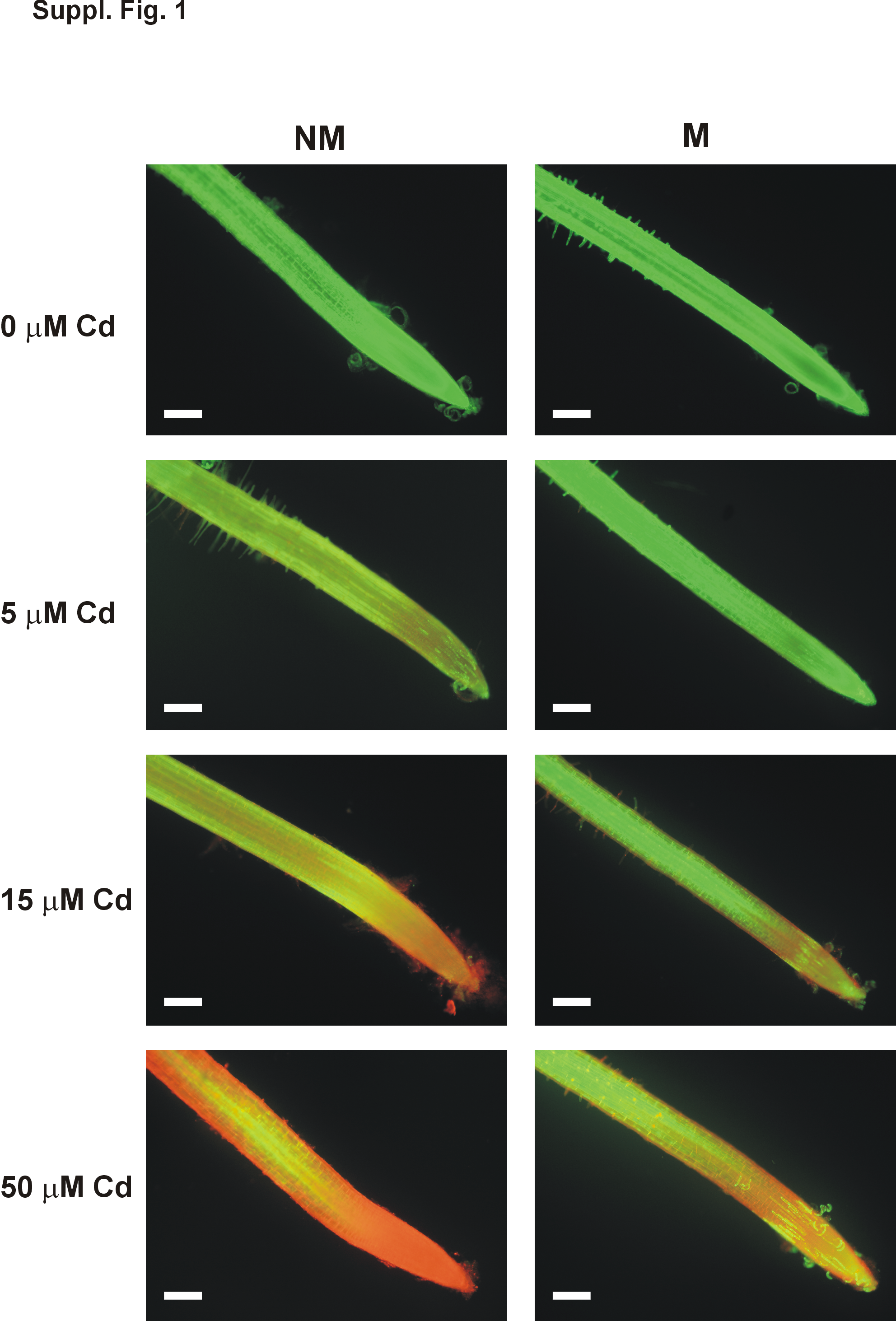

Supplement: Supplementary file 1 — (TIFF 17013 kb) Root viability of metallicolous (M) and nonmetallicolous (NM) plants of D. carthusianorum exposed to varying Cd concentrations for 14 days. Images of roots incubated in the mixture of FDA-PI were taken under fluorescent microscope at lens magnification 4x. Green fluorescence (metabolized FDA) and red fluorescence (PI) indicates viable and dead cells, respectively. Scale bars - 200 μm [file 11356_2014_3963_MOESM1_ESM.tif]

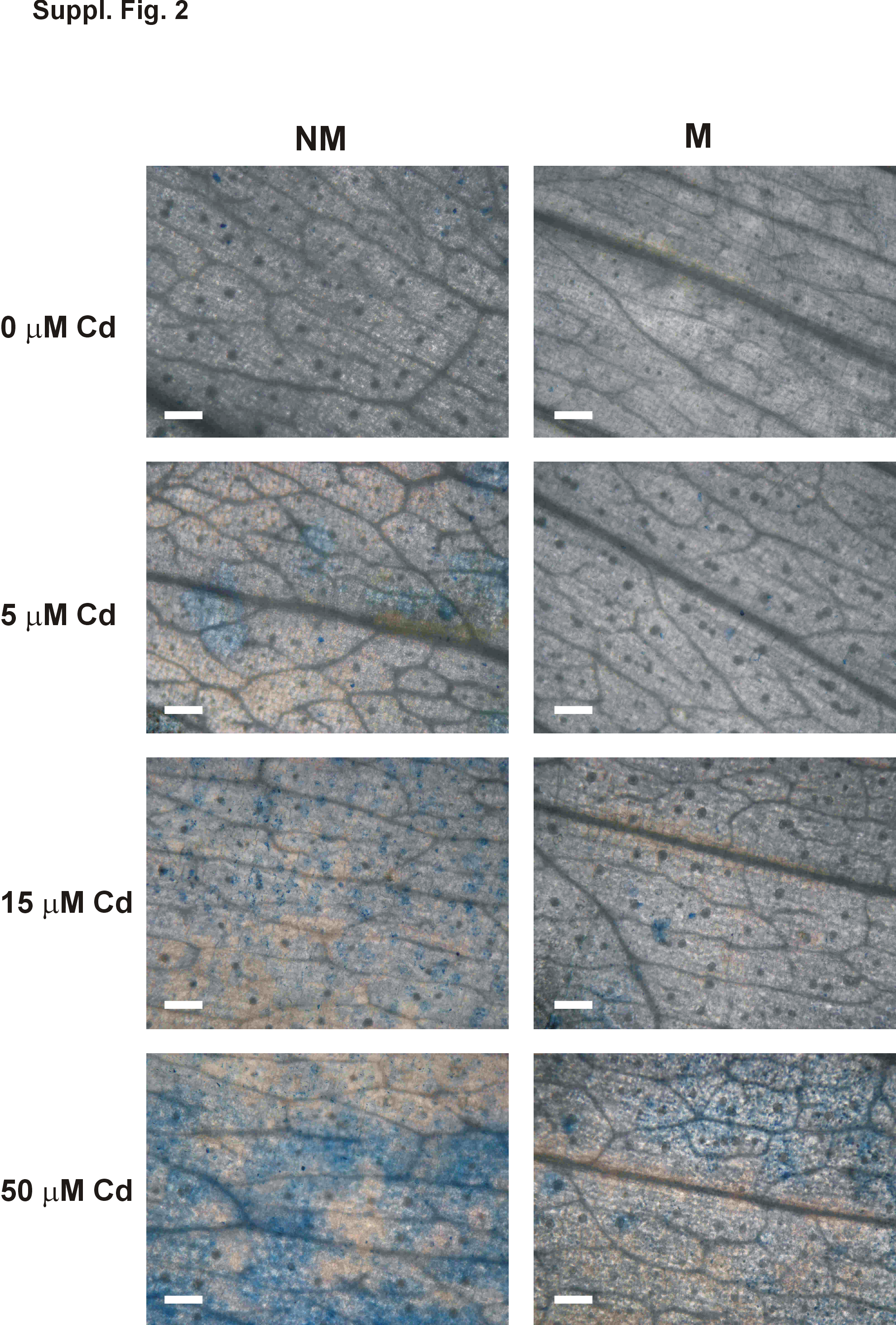

Supplement: Supplementary file 2 — (TIFF 16961 kb) Leaf viability of metallicolous (M) and nonmetallicolous (NM) plants of D. carthusianorum exposed to varying Cd concentrations for 14 days. Images of leaves stained in trypan blue were taken under light microscope at lens magnification 4×. Blue colour indicates cells with damaged plasma membranes. Scale bars - 200 μm [file 11356_2014_3963_MOESM2_ESM.tif]
